# Analysis of the genome content of *Lactococcus garvieae *by genomic interspecies microarray hybridization

**DOI:** 10.1186/1471-2180-10-79

**Published:** 2010-03-16

**Authors:** Mónica Aguado-Urda, Guillermo H López-Campos, José F Fernández-Garayzábal, Fernando Martín-Sánchez, Alicia Gibello, Lucas Domínguez, María M Blanco

**Affiliations:** 1 Departamento de Sanidad Animal, Facultad de Veterinaria, Universidad Complutense, 28040 Madrid, Spain; 2 Área de Bioinformática y Salud Pública, Instituto de Salud Carlos III, 28220 Madrid, Spain; 3 Centro de Vigilancia Sanitaria Veterinaria (VISAVET), Facultad de Veterinaria, Universidad Complutense, 28040 Madrid, Spain

## Abstract

**Background:**

*Lactococcus garvieae *is a bacterial pathogen that affects different animal species in addition to humans. Despite the widespread distribution and emerging clinical significance of *L. garvieae *in both veterinary and human medicine, there is almost a complete lack of knowledge about the genetic content of this microorganism. In the present study, the genomic content of *L. garvieae *CECT 4531 was analysed using bioinformatics tools and microarray-based comparative genomic hybridization (CGH) experiments. *Lactococcus lactis *subsp. *lactis *IL1403 and *Streptococcus pneumoniae *TIGR4 were used as reference microorganisms.

**Results:**

The combination and integration of *in silico *analyses and *in vitro *CGH experiments, performed in comparison with the reference microorganisms, allowed establishment of an inter-species hybridization framework with a detection threshold based on a sequence similarity of ≥ 70%. With this threshold value, 267 genes were identified as having an analogue in *L. garvieae*, most of which (n = 258) have been documented for the first time in this pathogen. Most of the genes are related to ribosomal, sugar metabolism or energy conversion systems. Some of the identified genes, such as *als *and *mycA*, could be involved in the pathogenesis of *L. garvieae *infections.

**Conclusions:**

In this study, we identified 267 genes that were potentially present in *L. garvieae *CECT 4531. Some of the identified genes could be involved in the pathogenesis of *L. garvieae *infections. These results provide the first insight into the genome content of *L. garvieae*.

## Background

*Lactococcus garvieae *is one of the most important bacterial pathogens that affect different farmed fish species in many countries, although its major impact is on the trout farm industry [1,2]. In addition to farmed fish, this microorganism has also been isolated from a wide range of wild fish species, from both fresh and marine water, as well as from giant fresh water prawns [3] and from wild marine mammals [4]. The host range of *L. garvieae *is not limited to aquatic species. This agent has also been identified in cows and water buffalos with subclinical mastitis [5,6] and from cat and dog tonsils [7]. In humans it has been isolated from the urinary tract, blood, and skin and from patients with pneumonia, endocarditis or septicaemia [8-11]. Recently, intestinal disorders in humans have been associated with the consumption of raw fish contaminated with this pathogen [12], which suggests that *L. garvieae *could be considered as a potentially zoonotic bacterium [3,12]. Despite the widespread distribution and emerging clinical significance of *L. garvieae *in both veterinary and human medicine, there is almost a complete lack of knowledge about the genetic content of this microorganism.

In the last few years, research in microbial genetics has changed fundamentally, from an approach involving the characterization of individual genes to a global analysis of microbial genomes. The availability of complete genome sequences has enabled the development of high-throughput nucleic acid hybridization technologies including macro- and microarrays. Microarrays have the capacity to monitor the genome content of bacterial strains or species very rapidly. Although whole-genome sequencing is definitely a powerful method for genetics, it is still expensive and time consuming. As an alternative, comparative genomic hybridization (CGH) experiments based on microarrays have been used to facilitate comparisons of unsequenced bacterial genomes. Array-based CGH using genome-wide DNA microarrays is used commonly to determine the genomic content of bacterial strains [13,14], but also for inter-species comparisons [14-16]. In this case, microarrays of closely related microorganisms that have been fully sequenced must be available. The primary advantage of this microarray approach is that it allows the identification of a large number of genes that are potentially present in an organism without the need for sequencing genomes. The disadvantage of this approach is that it indicates only the genes that are common between the fully sequenced relative and the strain of interest; genes unique to the strain of interest remain unknown [15,17]. In the present work the genetic content of *L. garvieae *CECT 4531 was studied by a combination of *in silico *analysis and *in vitro *microarray CGH experiments, using open reading frame (ORF) microarrays of two bacteria closely related to *L. garvieae*, namely *Lactococcus lactis *subsp. *lactis *IL1403 and *Streptococcus pneumoniae *TIGR4 [18,19].

## Methods

### Bacterial strains, culture conditions and isolation of genomic DNA

*Lactococcus lactis *subsp. *lactis *IL1403 (kindly provided by M.P. Gaya, INIA, Madrid, Spain) and *Streptococcus pneumoniae *TIGR4 (purchased form the American Type Culture Collection) were used as the reference sequenced microorganisms. The test strain of *Lactococcus garvieae *used for the experiments was CECT 4531 (purchased from the Spanish Type Culture Collection). The *L. lactis *subsp. *lactis *IL1403 and *L. garvieae *CECT 4531 were grown statically at 28°C in BHI broth (bioMérieux, Marcy l'Etoile, France). The *S. pneumoniae *TIGR4 was grown statically at 37°C in Todd Hewitt broth (Oxoid, Basingstoke, Hampshire, England). Cells were grown until the late-exponential phase of growth (OD600~1.5-2) and harvested for isolation and purification of genomic DNA using the DNeasy Blood and Tissue kit (Qiagen, Hilden, Germany) according to the manufacturer's specifications. The DNA concentrations were determined spectrophotometrically.

### DNA labelling

Aliquots (1-2 μg) of genomic DNA from the three strains were labelled fluorescently with Cy3-dUTP or Cy5-dUTP (Perkin-Elmer, Foster City, CA, USA), depending on whether the strain was used as a test or reference microorganism in the CGH experiments, respectively. Each DNA aliquot was fragmented by sonication to obtain fragments from 400 to 1000 bp. Fragmented DNA was mixed with 5 μL 10× NEBlot labelling buffer containing random sequence octamer oligonucleotides (New England Biolabs, Ipswich, MA, USA) and water to a final volume of 43.5 μL. This mixture was denatured by heating at 95°C for 5 min and then cooled for 5 min at 4°C. After this denaturing step, the remaining components of the labelling reaction were added: 5 μL of 10 × dNTP labelling mix (1.2 mM each dATP, dGTP and dCTP in 10 mM Tris pH 8.0, 1 mM EDTA) (New England Biolabs, Ipswich, MA, USA), 1.5 μL of 1 mM Cy3-dUTP or Cy5-dUTP and 1.5 μL of 10 U/μL Klenow fragment (Fermentas Life Sciences, Glen Burnie, MD, USA). The labelling reactions were incubated overnight at 37°C and then stopped by adding 2.5 μL of 0.5 M EDTA. Labelled DNA was purified from unincorporated label using a Qiaquick PCR Cleanup kit (Qiagen, Hilden, Germany) and dried under vacuum. The final DNA concentration and quality, as well as the labelling quality, were determined using a NanoDrop (NanoDrop Techonologies, Wilmington, DE, USA).

### Array-based comparative genome hybridization (CGH)

The *L. lactis *subsp. *lactis *IL1403 and *S. pneumoniae *TIGR4 microarrays used for the CGH analysis were purchased from Eurogentec (Serain, Belgium). The *L. lactis *microarray contains 4608 spots: 2126 duplicated ORFs, 32 negative controls and 324 empty spots. The *S. pneumoniae *microarray contains 4608 spots: 2087 duplicated ORFs, 224 negative controls and 210 empty spots.

The CGH experiments were performed by means of competitive hybridizations using DNA of *L. lactis *subsp. *lactis *IL1403 or *S. pneumoniae *TIGR4, depending on the array, as positive controls. The DNAs to be hybridized on the same array were labelled with Cy3-dUTP and Cy5-dUTP, respectively. For each microarray hybridization reaction, aliquots (1-2 μg) of labelled genomic DNAs of the reference (labelled with Cy3) and test (labelled with Cy5) strains, were mixed in 45 μL EGT hybridization solution (Eurogentec, Serain, Belgium) and denatured at 65°C for 2 min. The hybridization mixture was then loaded onto a microarray slide, covered with a coverslip and incubated at 38°C overnight. Following hybridization, the slides were washed in 2 × SSC, 0.5% SDS for 5 min followed by a second wash step in 1 × SSC, 0.25% SDS for 5 min. Finally, slides were rinsed in 0.2 × SSC and dried by centrifugation.

The results presented herein represent a compilation of sixteen separate CGH experiments: *L. lactis *subsp. *lactis *IL1403 arrays (reference microorganism) were hybridized with *S. pneumoniae *TIGR4 (test microorganism) (n = 2); *S. pneumoniae *TIGR4 arrays (reference microorganism) were hybridized with *L. lactis *subsp. *lactis *IL1403 (test microorganism) (n = 2); *L. lactis *subsp. *lactis *IL1403 arrays (reference microorganism) were hybridized with *L. garvieae *CECT 4531 (test microorganism) (n = 8); *S. pneumoniae *TIGR4 arrays (reference microorganism) were hybridized with *L. garvieae *CECT 4531 (test microorganism) (n = 4). The data discussed in this publication have been deposited in NCBI's Gene Expression Omnibus [20] and are accessible through GEO Series accession number GSE19005. http://www.ncbi.nlm.nih.gov/geo/query/acc.cgi?acc=GSE19005.

### Data acquisition and analysis

The microarray was scanned after hybridization using a Scanarray HT microarray scanner (Perkin-Elmer). The signal intensity of the two fluors was determined using ImaGene software (BioDiscovery, El Segundo, CA, USA). Microarray data were analysed using ImaGene software, Microsoft Excel and an in-house designed and built Microsoft Access database [21]. Gene calling was based on a signal-to-noise ratio (SNR) >3 for each spot. After the CGH experiments, a gene was considered to show a positive result when it was present in at least three of the four CGH assays. In the case of the *L. garvieae *CECT 4531 hybridizations with the *L. lactis *subsp. *lactis *IL1403 arrays, it was necessary to perform a larger number of assays (n = 8), owing to the poor quality of one of the batches of arrays used. Thus, the criterion chosen to determine a positive result in this case was when the gene was present in at least five of the eight CGH assays.

### *In silico *sequence analysis

Sequence analyses were carried out to assess the performance of the inter-species CGH protocol. Using the BLAT [22] and BLAST [23] programs, the sequences of the *L. lactis *microarray probes were aligned with the *S. pneumoniae *genome sequence, and *vice-versa*. The BLAT search parameters were 90%, 80% and 70% sequence identity (BLAT90, BLAT80 and BLAT70) and a 100 bp minimum alignment length (owing to the fact that the length of the array probe was between 100 and 400 bp). Available *L. garvieae *sequences of the nine previously identified genes that were positive in the CGH were aligned with the *L. lactis *subsp. *lactis *IL1403 or *S. pneumoniae *TIGR4 genomes and with the sequences of the immobilized probes of these genes in the corresponding microarray using BLAST [23] and BLAST 2 sequences [24] programs.

## Results

### Inter-species comparison framework

*In silico *analyses were performed to compare the sequences of the immobilized probes in the microarray of each reference organism with the sequences of their complete genomes available in GenBank (*L. lactis *subsp. *lactis *IL1403: NC_002662 and *S. pneumoniae *TIGR4: NC_003028). The BLAT alignment of the *L. lactis *IL1403 probes on the *S. pneumoniae *TIGR4 genome allowed the identification of 1 ORF with BLAT90, 65 ORFs with BLAT80 and 159 ORFs with BLAT70. Moreover, the BLAT alignment of the probes represented on the *S. pneumoniae *microarray on the *L. lactis *genome demonstrated 1 ORF, 63 ORFs and 165 ORFs for BLAT90, BLAT80 and BLAT70, respectively.

The CGH experiments based on swapping off the microarrays between *S. pneumoniae *and *L. lactis *identified 65 common ORFs. To evaluate the accuracy of the microarray CGH experiments, we compared these results with those of the *in silico *analysis. Out of the 65 genes, 47 (72%) showed similarities greater than 80%, 16 genes (25%) exhibited a similarity between 70% and 80%, and only 2 genes (3%) showed a similarity slightly lower than 70% (66-68%) (Table 1). In summary, 97% of the genes detected by CGH showed similarities greater than 70% at the nucleotide level.

**Table 1:** *In silico *analysis of the common genes detected by CGH in the reference microorganisms

CGH positive gene ID^a^	BLAT80^b^	BLAT70^b, c^	% Identity in BLAST^d^
*atpD/SP1508*	++		
*dnaG/SP1072*	-	-	66%
*dnaJ/SP0519*	+		
*dnaK/SP0517*	++		
*enoA/SP1128*	++		
*fbaA/SP0605*	++		
*ftsZ/SP1666*	++		
*fusA/SP0273*	++		
*gapB/SP2012*	++		
*gltX/SP2069*	-	++	
*ldh/SP1220*	++		
*lepA/SP1200*	-	++	
*leuS/SP0254*	++		
*lysS/SP0713*	-	++	
*msmk/SP1580*	++		
*murA1/SP1081*	-	+	
*pepC/SP0281*	+		
*pfk/SP0896*	-	+	
*pgiA/SP2070*	+		
*pgk/SP0499*	++		
*pmg/SP1655*	++		
*ptnAB/SP0284*	+		
*ptnD/SP0282*	-	+	
*ptsI/SP1176*	-	++	
*purA/SP0019*	-	-	76%
*pyk/SP0897*	++		
*rplA/SP0631*	++		
*rplB/SP0212*	++		
*rplC/SP0209*	++		
*rplF/SP0225*	+		
*rplJ/SP1355*	+		
*rplK/SP0630*	++		
*rplL/SP1354*	++		
*rplM/SP0294*	++		
*rplN/SP0219*	++		
*rplO/SP0229*	++		
*rplQ/SP0237*	+		
*rplS/SP1293*	++		
*rplV/SP0214*	+		
*rpmI/SP0960*	+		
*rpmJ/SP0233*	++		
*rpoB/SP1961*	-	+	
*rpoC/SP1960*	+	++	
*rpoD/SP1073*	++		
*rpsA/SP0862*	-	+	
*rpsB/SP2215*	-	++	
*rpsC/SP0215*	++		
*rpsD/SP0085*	++		
*rpsE/SP0227*	++		
*rpsG/SP0272*	++		
*rpsJ/SP0208*	++		
*rpsK/SP0235*	++		
*rpsL/SP0271*	++		
*rpsM/SP0234*	+		
*rpsP/SP0775*	+		
*rpsS/SP0213*	+		
*topA/SP1263*	-	+	
*tuf/SP1489*	++		
*typA/SP0681*	++		
*upp/SP0745*	-	-	77%
*ychH/SP2097*	+		
*yciA/SP2096*	-	-	68%
*yjiF/SP1565*	-	+	
*yjkI/SP2209*	-	+	
*yyaL/SP0004*	++		

^a ^Reference organisms gene ID: *Lactococcus lactis *subsp. *lactis *IL1403/*Streptococcus pneumoniae *TIGR4

^b ^++ Genes detected in both alignments, *L. lactis *subsp. *lactis *IL1403 array probes vs *S. pneumoniae *TIGR4 genome, and *S. pneumoniae *TIGR4 array probes vs *L. lactis *subsp. *lactis *IL1403 genome; + positive in one of the two cases.

^c ^Only the results for the negative genes in BLAT80 are shown.

^d ^Only the results for the negative genes in both BLAT80 and BLAT70 are shown.

After combined analysis of the results obtained *in silico *and *in vitro*, we established, under the hybridization conditions used in this study, a detection threshold based on a sequence similarity of ≥ 70% for alignments longer than 100 bp. This was established as the reference framework for the inter-species CGH assays.

### *In vitro *microarray CGH experiments with *L. garvieae *CECT 4531 vs reference microorganisms *L. lactis *subsp. *lactis *IL1403 and *S. pneumoniae *TIGR4, and *in silico *analysis of available sequences from *L. garvieae*

The microarray CGH experiments identified 267 genes in *L. garvieae *that had analogues in *L. lactis *and/or *S. pneumoniae *(Additional file 1). Of these, 111 genes (41.6%) were identified only with the *L. lactis *microarray, 70 genes (26.2%) only with the microarray of *S. pneumoniae*, and 86 genes (32.2%) were identified with both microarrays. These genes belong to diverse functional groups (Table 2). Most of the genes (96.6%) have been documented for the first time in *L. garvieae*. Only nine genes (four present in both reference microorganisms: *atpD/SP1508, pfk/SP0896, tig/SP0400, tuf/SP1489*; three present in *L. lactis*: *als, ddl, galK*; two present in *S. pneumoniae: SP0766, SP1219*) out of the 267 genes detected have been either identified or sequenced before in diverse strains of *L. garvieae *(Tables 3 and 4). *In silico *analysis of these previously sequenced genes (n = 9) of *L. garvieae *were performed to assess the efficacy of the methodology. Alignments of these available sequences with the genomes of the corresponding reference microorganism and their respective array probes showed nucleotide identities ranging between 70% and 86% (Tables 3 and 4). Most of the available sequences (80%) showed similarities greater than 75%.

**Table 2:** Functional groups of genes identified in *L. garvieae *CECT 4531 according to the COG database

Functional Group	Homologous in *L. lactis *subsp. *lactis *IL1403	Homologous in *S. pneumoniae *TIGR4
Amino acid transport and metabolism	14	10
Carbohidrate transport and metabolism	24	15
Cell cycle control, cell division, cromosome partitioning	4	2
Cell wall/membrane/envelope biogenesis	5	4
Coenzime transport and metabolism	1	1
DNA replication, recombination and repair	8	12
Energy production and conversion	11	6
Inorganic ion transport and metabolism	4	5
Intracellular trafficking, secretion and vesicular transport	4	2
Lipid transport and metabolism	2	0
Nucleotide transport and metabolism	15	11
Phage capside proteins	1	0
Post translational modification, protein turnover, chaperones	8	8
Signal transduction mechanisms	2	3
Transcription	7	6
Translation, ribosomal structure and biogenesis	64	60
Unknown function	23	11
**Total**	**197**	**156**

**Table 3:** *In silico *analysis of the available sequences of the genes detected in *L. garvieae *by CGH

Gene ID	GenBank accession number of *L. garvieae *sequence	*L. garvieae *strain	Similarity with *L. lactis *subsp. *lactis *IL1403 gene (%)	Similarity with array probe (%)
*als*	EF450031	UNIUD074	77	76
*atpD*	AX111128	from patent WO0123604	86	86
*ddl*	AF170808	*E. serolicida*	72	75
*galK*	EU153555	DSM 20684	78	79
*pfk*	AB024532	SA8201	85	84
*tig*	AB024531	SA8201	82	-
*tuf*	AX109994	from patent WO0123604	80	77

Results for the *L. lactis *subsp. *lactis *IL1403 array based-CGH

**Table 4:** *In silico *analysis of the available sequences of the genes detected in *L. garvieae *by CGH

Gene ID	GenBank accession number of *L. garvieae *sequence	*L. garvieae *strain	Similarity with *S. pneumoniae *TIGR4 gene (%)	Similarity with array probe (%)
*SP1508*	AX111128	from patent WO0123604	82	82
*SP0896*	AB024532	SA8201	80	79
*SP0766*	AM490328	JIP 31-90 (2)	71	79
	AJ387925	CIP 102507 T	70	70
	AJ387923	*E. serolicida *ATCC49156	70	70
*SP04000*	AB024531	SA8201	74	-
*SP1489*	AX109994	from patent WO0123604	80	79
*SP1219*	AB364641	20-92	84	86
	AB364640	Lc.1236	84	85
	AB364639	Lc. 925	85	85
	AB364638	Lc. 881	84	84
	AB364637	Lc. 337	85	85
	AB364633	LMG9472	85	85
	AB364632	ATCC43921	84	84
	AB364627	G50202	84	84
	AB364626	KGLA5224	84	84
	AB364625	EH5803	83	83
	AB364624	KG9408	84	84

Results for the *S. pneumoniae *TIGR4 array based-CGH

## Discussion

In the present study, commercial microarrays of *L. lactis *subsp. *lactis *IL1403 and *S. pneumoniae *TIGR4 were used to determine the presence of homologous genes in *L. garvieae*. Both *L. lactis *and *S. pneumoniae *were chosen as reference organisms because they are closely related to *L. garvieae *[18,19] and their genomes have been fully sequenced. Although these CGH experiments cannot detect and identify genes that are likely to exist only in the target microorganism, this approach reveals genes that are common to both the reference and the target organisms, allowing the identification of a large number of genes potentially present in an organism without the need for sequencing genomes [17,25].

In experiments that involve inter-species comparison it is necessary to establish a framework that allows accurate comparison and interpretation of the results. Thus, the first efforts were focused on establishing that framework by the combination and integration of *in silico *analyses and *in vitro *microarray CGH experiments to compare the reference organisms *L. lactis *subsp. *lactis *IL1403 and *S. pneumoniae *TIGR4. Signal intensity has been used to assess the level of similarity between two genes in inter-species CGH experiments [15]. However, this approach may be influenced, and therefore biased, by different factors, such as regional sample labelling effects, probe accessibility or local hybridization issues [13]. For these reasons, in the present study signal intensity was not considered for determining whether a gene was positive or not in the inter-species CGH experiments.

These analyses revealed that nearly all the genes common to *L. lactis *and *S. pneumoniae *that were detected by swap microarray CGH experiments (97%) exhibited a sequence similarity of at least 70% (Table 1). Only two genes (*dnaG *and *yciA*) detected in the microarray CGH experiments showed a sequence similarity slightly lower than 70% (66 and 68%, respectively; Table 1). Variability in the factors that influence the CGH signals, such as systematic errors (e.g. dye effects), copy number variation, and sequence divergence between the analysed samples [13], may explain these results. The comparison of the results of both analyses, *in silico *and *in vitro*, for the reference microorganisms (Table 1) allowed us to establish that, under our experimental conditions, it was possible to detect and identify inter-species hybridization with a detection threshold based on a sequence similarity of ≥70%.

Therefore, our threshold value of sequence similarity ≥70% was set up directly from the comparison of the results of the *in silico *and *in vitro *analyses of the present study. This threshold value was used subsequently to interpret the results of the microarray-based CGH experiments comparing *L. garvieae *and the reference microorganisms. Less stringent hybridization conditions would probably have allowed the identification of a larger number of genes, but this would have also resulted in lower specificity. Given that the final aim of the experiment was the identification of genes potentially present in *L garvieae*, it was preferred to maintain stringent hybridization conditions, therefore increasing the specificity and the reliability of the results. Hence, the genes detected in the CGH experiments should have an analogue in *L. garvieae *with a nucleotide sequence identity greater than 70% with the respective gene in the reference organism.

The CGH hybridizations using *L. lactis *subsp. *lactis *IL1403 and *S. pneumoniae *TIGR4 microarrays identified 267 analogous genes in *L. garvieae *(Additional file 1). Only 3.4% of these genes (nine out of 267) have been characterized or sequenced previously by other groups in different strains of *L. garvieae *[[18,26-29], and GenBank sequences: AX109994, AB364624, AB364625, AB364626, AB364627, AB364632, AB364633, AB364637, AB364638, AB364639, AB364640, AB364641, EU153555]. The alignments of the available sequences of these nine previously of these nine previously identified genes in *L. garvieae *with both the sequences of these genes from the reference microorganisms and those from the array probe showed nucleotide similarities greater than 70% (70-86%) between them (Tables 3 and 4). These data are consistent with the detection threshold value discussed previously. Therefore it is reasonable to assume that the other genes detected in *L. garvieae *CECT 4531 by CGH experiments will also have at least 70% sequence similarity with the respective genes in the reference microorganisms. The positive result obtained in both CGH experiments for the *tig/SP0400 *gene (Tables 3 and 4), was unexpected given the absence of similarity between the available sequence and the probes on both microarrays. This result could be explained by the fact that the available sequence for *L. garvieae *is partial, and it represents a part of the gene that does not correspond with the probe.

We classified the ORFs into clusters of orthologous genes (COGs) [30]. The 267 genes identified in *L. garvieae *CECT 4531 (Additional file 1) belong to diverse biological functional groups (Table 2). Most of the genes detected in *L. garvieae *(about 66%) were related to meaningful biological functions such as those related to ribosomal functions, sugar metabolism or energy conversion systems, which are usually represented in *Lactobacillales *[31]. The remaining genes identified included "housekeeping genes", such as *gyrB*, *sodA*, *recA*, *ileS*, *rpoD*, *dnaK *and *ddl *[19], genes of diverse functional groups and genes with unknown functions. Some of them are of interest because they could be involved in the pathogenesis of *L. garvieae *infections. For example, the gene *als*, which has been described as an important factor for host colonization by El Tor biotypes of *Vibrio cholerae *[32], has also been suggested to be one of the genes required for survival of *L. garvieae *in fish [27]. In addition, the gene *mycA*, which was detected for the first time in *L. garvieae *in the present study, encodes an antigen that cross-reacts with myosin, and members of this family of proteins have been suggested to play an important role in the pathogenesis of streptococcal infections [33].

Sequencing of the genes identified in this work is beyond the scope of this initial study, but the data provided can be the starting point for future genetic analysis of *L. garvieae *strains from different ecological niches or adapted to different host species.

This study provides the first insight into the genome content of *L. garvieae *and suggests that CHG could be a useful approach for studying the genetic content of other Gram-positive catalase-negative cocci of human and veterinary relevance.

## Conclusions

In the present work, a comparative analysis based on microarray interspecies hybridization and on the use of bioinformatic tools was used for the first time to study the genetic content of *L. garvieae *CECT 4531. It is important to remark that the integration of results from bioinformatics and microarray-based CGH requires the definition of a framework that allows an accurate comparison and interpretation of the results obtained. Once this framework was established, it was possible to identify 267 genes potentially present in *L. garvieae *CECT 4531. Some of the identified genes, such as the *als *and *mycA *genes, could be involved in the pathogenesis of *L. garvieae *infections.

In summary, these results provide the first insight into the genome content of *L. garvieae *and could be useful for future understanding of the genetics of this pathogenic microorganism.

## Authors' contributions

MAU carried out the microarray experiments and the bioinformatics analyses, and participated in the analysis of the data and drafting of the manuscript. GHLC designed the microarray experiments, and participated in analysis of the data and drafting of the manuscript. JFFG participated in and supervised drafting of the manuscript. FMS participated in and supervised the design of the microarray experiments and the analysis of the microarray data. AG participated in the design of the study and drafting of the manuscript. LD participated in the design of the study. MB conceived the study, and participated in its design and coordination. All the authors read and approved the final manuscript.

## Supplementary Material

Additional file 1 Genes potentially identified in *L. garvieae *CECT 4531 and their homologues in *L. lactis *subsp. *lactis *IL1403 and *S. pneumoniae *TIGR4. Click here for file
