# Supplementary material for: Analysis of the genome content of Lactococcus garvieae by genomic interspecies microarray hybridization
Source: BMC Microbiol. 2010 Mar 16;10:79. doi: 10.1186/1471-2180-10-79 (PMC2851595; doi:10.1186/1471-2180-10-79)
Supplement: Additional file 1 — Genes potentially identified in L. garvieae CECT 4531 and their homologues in L. lactis subsp. lactis IL1403 and S. pneumoniae TIGR4. [file 1471-2180-10-79-S1.DOC]

**Additional file 1** – **Genes potentially identified in *L. garvieae* CECT 4531 and their homologues in *L. lactis* subsp*. lactis* IL1403 and *S. pneumoniae* TIGR4**

| Identified genes in *L. garvieae* CECT 4531 | *L. lactis* subsp*. lactis* IL1403 homologous gene ID | *S. pneumoniae* TIGR4 homologous gene ID |
| --- | --- | --- |
| Ornithine carbamoyltransferase (EC 2.1.3.3) | *arcB* | *SP2150* |
| ATP synthase beta subunit (EC 3.6.1.34)^a^ | *atpD* | *SP1508* |
| Phospho-beta-glucosidase | *bglA* | *SP0303* |
| DnaJ protein | *dnaJ* | *SP0519* |
| DnaK protein | *dnaK* | *SP0517* |
| Elongation factor P | *efp* | *SP0435* |
| Enolase (EC 4.2.1.11) | *enoA* | *SP1128* |
| Fructose-bisphosphate aldolase (EC 4.1.2.13) | *fbaA* | *SP0605* |
| Signal recognition particle protein Ffh | *ffh* | *SP1287* |
| Cell division protein FtsZ | *ftsZ* | *SP1666* |
| Elongation factor G | *fusA* | *SP0273* |
| Glyceraldehyde 3-phosphate dehydrogenase (EC 1.2.1.12) | *gapB* | *SP2012* |
| Glycerol uptake facilitator | *glpF2* | *SP2184* |
| Glycerol kinase (EC 2.7.1.30) | *glpK* | *SP2186* |
| Guanylate kinase (EC 2.7.4.8) | *gmk* | *SP1738* |
| Decarboxylating 6-phosphogluconate dehydrogenase | *gnd* | *SP0375* |
| Glycerol-3-phosphate dehydrogenase (EC 1.1.1.94) | *gpdA* | *SP2091* |
| Translation initiation factor IF-1 | *infA* | *SP0232* |
| GTP-binding protein LepA | *lepA* | *SP1200* |
| Leucyl-tRNA synthetase (EC 6.1.1.4) | *leuS* | *SP0254* |
| Lysyl-tRNA synthetase (EC 6.1.1.6) | *lysS* | *SP0713* |
| Topoisomerase IV subunit A (EC 5.99.1.-) | *parC* | *SP0855* |
| 6-phosphofructokinase (EC 2.7.1.11)^a^ | *pfk* | *SP0896* |
| Glucose-6-phosphate isomerase A (EC 5.3.1.9) | *pgiA* | *SP2070* |
| Phosphoglycerate kinase (EC 2.7.2.3) | *pgk* | *SP0499* |
| Phosphoglycerate mutase (EC 5.4.2.1) | *pmg* | *SP1655* |
| Spermidine/putrescine ABC transporter ATP-binding protein | *potA* | *SP1387* |
| Ribose-phosphate pyrophosphokinase (EC 2.7.6.1) | *prsA* | *SP0027* |
| Phosphate ABC transporter ATP binding protein | *pstA* | *SP1396* |
| Phosphate ABC transporter permease protein | *pstC* | *SP1399* |
| Mannose-specific PTS system component IIAB (EC 2.7.1.69) | *ptnAB* | *SP0284* |
| Phosphoenolpyruvate-protein phosphotransferase (EC 2.7.3.9) | *ptsI* | *SP1176* |
| Phosphocarrier protein Hpr | *ptsH* | *SP1177* |
| Adenylosuccinate lyase (EC 4.3.2.2) | *purB* | *SP0056* |
| Pyruvate kinase (EC 2.7.1.40) | *pyk* | *SP0897* |
| Pyrimidine operon regulator | *pyrR* | *SP1278* |
| RecA protein | *recA* | *SP1940* |
| ATP-dependent RNA helicase | *rheA* | *SP1586* |
| 50S ribosomal protein L1 | *rplA* | *SP0631* |
| 50S ribosomal protein L2 | *rplB* | *SP0212* |
| 50S ribosomal protein L3 | *rplC* | *SP0209* |
| 50S ribosomal protein L6 | *rplF* | *SP0225* |
| 50S ribosomal protein L10 | *rplJ* | *SP1355* |
| 50S ribosomal protein L11 | *rplK* | *SP0630* |
| 50S ribosomal protein L7/L12 | *rplL* | *SP1354* |
| 50S ribosomal protein L14 | *rplN* | *SP0219* |
| 50S ribosomal protein L15 | *rplO* | *SP0229* |
| 50S ribosomal protein L17 | *rplQ* | *SP0237* |
| 50S ribosomal protein L18 | *rplR* | *SP0226* |
| 50S ribosomal protein L19 | *rplS* | *SP1293* |
| 50S ribosomal protein L20 | *rplT* | *SP0961* |
| 50S ribosomal protein L22 | *rplV* | *SP0214* |
| 50S ribosomal protein L27 | *rpmA* | *SP1107* |
| 50S ribosomal protein L28 | *rpmB* | *SP0441* |
| 50S ribosomal protein L30 | *rpmD* | *SP0228* |
| 50S ribosomal protein L31 | *rpmE* | *SP1299* |
| 50S ribosomal protein L32 | *rpmF* | *SP2134* |
| 50S ribosomal protein L35 | *rpmI* | *SP0960* |
| DNA-directed RNA polymerase beta chain (EC 2.7.7.6) | *rpoB* | *SP1961* |
| DNA-directed RNA polymerase beta' chain (EC 2.7.7.6) | *rpoC* | *SP1960* |
| 30S ribosomal protein S1 | *rpsA* | *SP0862* |
| 30S ribosomal protein S2 | *rpsB* | *SP2215* |
| 30S ribosomal protein S3 | *rpsC* | *SP0215* |
| 30S ribosomal protein S4 | *rpsD* | *SP0085* |
| 30S ribosomal protein S5 | *rpsE* | *SP0227* |
| 30S ribosomal protein S7 | *rpsG* | *SP0272* |
| 30S ribosomal protein S10 | *rpsJ* | *SP0208* |
| 30S ribosomal protein S11 | *rpsK* | *SP0235* |
| 30S ribosomal protein S12 | *rpsL* | *SP0271* |
| 30S ribosomal protein S13 | *rpsM* | *SP0234* |
| 30S ribosomal protein S16 | *rpsP* | *SP0775* |
| 30S ribosomal protein S17 | *rpsQ* | *SP0218* |
| 30S ribosomal protein S19 | *rpsS* | *SP0213* |
| 30S ribosomal protein S21 | *rpsU* | *SP1414* |
| preprotein translocase SecA subunit | *secA* | *SP1702* |
| Theronyl-tRNA synthetase (EC 6.1.1.3) | *thrS* | *SP1631* |
| Trigger factor^a^ | *tig* | *SP0400* |
| Elongation factor Ts | *tsf* | *SP2214* |
| Elongation factor Tu^a^ | *tuf* | *SP1489* |
| GTP-binding protein TypA/BipA | *typA* | *SP0681* |
| Valyl-tRNA synthetase (EC 6.1.1.9) | *valS* | *SP0568* |
| ABC transporter ATP binding protein | *yahG* | *SP2230* |
| Conserved hypothetical protein | *yhcI* | *SP0020* |
| GTP-binding protein | *yphL* | *SP1709* |
| Protease | *yugD* | *SP1429* |
| GTP-binding protein | *yyaL* | *SP0004* |
| Acetate kinase (EC 2.7.2.1) | *ackA2* |  |
| Alpha-acetolactate synthase^a^ | *als* |  |
| Arginine deiminase (EC 3.5.3.6) | *arcA* |  |
| Chorismate synthase (EC 4.6.1.4) | *aroC* |  |
| Cellobiose-specific PTS system IIC component (EC 2.7.1.69) | *celB* |  |
| ClpB protein | *clpB* |  |
| Cardiolipin synthase | *clsB* |  |
| Transcriptional regulator | *codY* |  |
| D-alanine-D-alanine ligase (EC 6.3.2.4)^a^ | *ddl* |  |
| ABC transporter ATP binding protein | *ecsA* |  |
| 3R-hydroxymyristoyl-acyl carrier protein dehydratase (EC 4.2.1.1) | *fabZ2* |  |
| Cell division protein FtsA | *ftsA* |  |
| Cell-division ATP-binding protein FtsE | *ftsE* |  |
| Cell division protein FtsH | *ftsH* |  |
| Galactokinase (EC 2.7.1.6)^a^ | *galK* |  |
| Glutamine ABC transporter ATP-binding protein | *glnQ* |  |
| Glycerol-3-phosphate dehydrogenase (EC 1.1.99.5) | *glpD* |  |
| Glutamyl-tRNA synthetase (EC 6.1.1.17) | *gltX* |  |
| Glycyl-tRNA synthetase alpha chain (EC 6.1.1.14) | *glyS* |  |
| IMP dehydrogenase (EC 1.1.1.205) | *guaB* |  |
| Histidyl-tRNA synthetase (EC 6.1.1.21) | *hisS* |  |
| HU like DNA-binding protein | *hslA* |  |
| Isoleucyl-tRNA synthetase (EC 6.1.1.5) | *ileS* |  |
| Dihydroxynaphthonic acid synthase (EC 4.1.3.36) | *menB* |  |
| Methyonyl-tRNA synthetase (EC 6.1.1.10) | *metS* |  |
| UDP-N-acetylglucosamine 1-carboxyvinyltransferase | *murA1* |  |
| A/G-specific adenine glycosylase (EC 3.2.2.1) | *mutY* |  |
| Myosin-crossreactive antigen | *mycA* |  |
| Glucosamine-6-P isomerase (EC 5.3.1.10) | *nagB* |  |
| NADH dehydrogenase | *noxB* |  |
| Transcription termination protein NusA | *nusA* |  |
| Oligopeptide ABC trasporter ATP binding protein | *optD* |  |
| PDH E1 component alpha subunit (EC 1.2.4.1) | *pdhA* |  |
| PDH E1 component beta subunit (EC 1.2.4.1) | *pdhB* |  |
| Dihydrolipoamide acetyltransferase component of PDH complex (EC 2.3.1.12*)* | *pdhC* |  |
| Lipoamide dehydrogenase component of PDH complex (EC 1.8.1.4) | *pdhD* |  |
| Glutamyl aminopeptidase | *pepA* |  |
| Dipeptidase | *pepDB* |  |
| Methionine aminopeptidase | *pepM* |  |
| Aminopeptidase N | *pepN* |  |
| Tripeptidase | *pepT* |  |
| Pyruvate-formate lyase (EC 2.3.1.54) | *pfl* |  |
| 5'-methylthioadenosine/S-adenosylhomocysteine nucleosidase (EC 3.2.2.9) | *pfs* |  |
| Phenylalanil-tRNA synthetase alpha chain (EC 6.1.1.20) | *pheS* |  |
| Prophage pi3 protein 22, major head protein precursor | *pi322* |  |
| Peptidyl-prolyl cis-trans isomerase | *ppiB* |  |
| Peptide chain release factor 2 | *prfB* |  |
| Prophage ps1 protein 15, transcriptional regulator | *ps115* |  |
| Phosphate ABC transporter substrate binding protein | *pstE* |  |
| Phosphate ABC transporter substrate binding protein | *pstF* |  |
| Beta-glucoside-specific PTS system IIABC component (EC 2.7.1.69) | *ptbA* |  |
| Cellobiose-specific PTS system IIB component (EC 2.7.1.69) | *ptcB* |  |
| Peptidyl-tRNA hydrolase (EC 3.1.1.29) | *pth* |  |
| Mannose-specific PTS system component IIC (EC 2.7.1.69) | *ptnC* |  |
| Mannose-specific PTS system component IID (EC 2.7.1.69) | *ptnD* |  |
| Bifunctional purine biosynthesis protein PurH | *purH* |  |
| Phosphoribosylformylglycinamidine synthase II (EC 6.3.5.3) | *purL* |  |
| Regulator of purine biosynthetic genes | *purR* |  |
| CTP synthetase | *pyrG* |  |
| UMP-kinase (EC 2.7.4.-) | *pyrH* |  |
| Uracil permease | *pyrP* |  |
| ppGpp synthetase I (EC 2.7.6.5) | *relA* |  |
| Polysaccharide ABC transporter permease protein | *rgpC* |  |
| Glucose-1-phosphate thymidylyltransferase (EC 2.7.7.24) | *rmlA* |  |
| dTDP-glucose 4,6-dehydratase | *rmlB* |  |
| 50S ribosomal protein L21 | *rplU* |  |
| 50S ribosomal protein L24 | *rplX* |  |
| 50S ribosomal protein L34 | *rpmH* |  |
| DNA-directed RNA polymerase alpha chain (EC 2.7.7.6) | *rpoA* |  |
| Major RNA polymerase sigma factor | *rpoD* |  |
| 30S ribosomal protein S6 | *rpsF* |  |
| 30S ribosomal protein S8 | *rpsH* |  |
| 30S ribosomal protein S9 | *rpsI* |  |
| 30S ribosomal protein S14 | *rpsN* |  |
| 30S ribosomal protein S18 | *rpsR* |  |
| 30S ribosomal protein S20 | *rpsT* |  |
| Preprotein translocase SecY subunit | *secY* |  |
| Single-strand binding protein | *ssbB* |  |
| Queuine tRNA-ribosyltransferase (EC 2.4.2.29) | *tgt* |  |
| GTP-binding protein ThdF | *thdF* |  |
| Thioredoxin reductase (EC 1.6.4.5) | *trxB1* |  |
| Uridine kinase (EC 2.7.1.48) | *udk* |  |
| Uracil phosphoribosyltransferase (EC 2.4.2.9) | *upp* |  |
| Excinuclease ABC subunit A | *uvrA* |  |
| Xanthine phosphoribosyltransferase (EC 2.4.2.-) | *xpt* |  |
| Hypothetical protein | *yaiA* |  |
| Hypothetical protein | *ybcG* |  |
| Hypothetical protein | *ybdD* |  |
| Amidase | *ybgE* |  |
| Hypothetical protein | *ybjJ* |  |
| Hypothetical protein | *ybjK* |  |
| Hypothetical protein | *ycfD* |  |
| Hypothetical protein | *yciH* |  |
| Hypothetical protein | *ydiB* |  |
| Diadenosine 5',5'''-P1,P4-tetraphosphate hydrolase | *yffD* |  |
| Hypothetical protein | *ygdA* |  |
| Hypothetical protein | *yhfC* |  |
| Oxidoreductase | *yiaB* |  |
| Hypothetical protein | *yibB* |  |
| Amino acid permease | *yibG* |  |
| Amino acid ABC transporter ATP binding protein | *yjgE* |  |
| Conserved hypothetical protein | *yjiF* |  |
| Hypothetical protein | *ykjI* |  |
| Hypothetical protein | *yleC* |  |
| Cation transport ATPase | *yqgG* |  |
| Hypothetical PROTEIN | *yriD* |  |
| Hypothetical PROTEIN | *ysbD* |  |
| Conserved hypothetical protein | *yseI* |  |
| Hypothetical protein | *yteB* |  |
| Transporter | *yvdD* |  |
| Unknown protein | *ywaB* |  |
| Adenylosuccinate synthetase |  | *SP0019* |
| Type II DNA modification methyltransferase Spn5252 |  | *SP0023* |
| Membrane protein |  | *SP0034* |
| Phosphoribosylaminoimidazole carboxylase, | *SP0053* | *SP0053* |
| ABC transporter, substrate-binding protei | *SP0148* | *SP0148* |
| Ribosomal protein L4 | *SP0210* | *SP0210* |
| Ribosomal protein L16 | *SP0216* | *SP0216* |
| Ribosomal protein L5 | *SP0221* | *SP0221* |
| Ribosomal protein L36 | *SP0233* | *SP0233* |
| Holliday junction DNA helicase RuvB | *SP0259* | *SP0259* |
| Ribosomal protein L13 | *SP0294* | *SP0294* |
| Glutamyl-tRNA(Gln) amidotransferase, B subunit | *SP0436* | *SP0436* |
| Peptide chain release factor 3 | *SP0439* | *SP0439* |
| Formate acetyltransferase | *SP0459* | *SP0459* |
| Glutamine synthetase, type I | *SP0502* | *SP0502* |
| Heat shock protein GrpE | *SP0516* | *SP0516* |
| Glucokinase | *SP0668* | *SP0668* |
| ATP-dependent Clp protease, proteolytic subunit | *SP0746* | *SP0746* |
| ATP-dependent RNA helicase, DEAD/DEAH box | *SP0761* | *SP0761* |
| Superoxide dismutase, manganese-dependent^a^ | *SP0766* | *SP0766* |
| Conserved hypothetical protein | *SP0768* | *SP0768* |
| DNA gyrase subunit B | *SP0806* | *SP0806* |
| Lipoprotein | *SP0845* | *SP0845* |
| ABC transporter, ATP-binding protein | *SP0867* | *SP0867* |
| Aminotransferase, class-V | *SP0880* | *SP0880* |
| Thiazole biosynthesis protein ThiI | *SP0881* | *SP0881* |
| Transcriptional regulator, LysR family | *SP0927* | *SP0927* |
| Ribosome recycling factor | *SP0945* | *SP0945* |
| Translation initiation factor IF-3 | *SP0959* | *SP0959* |
| Multi-drug resistance efflux pump | *SP0972* | *SP0972* |
| O-methyltransferase | *SP0980* | *SP0980* |
| 5'-methylthioadenosine/S-adenosylhomocysteine nucleosidase | *SP0991* | *SP0991* |
| Serine hydroxymethyltransferase | *SP1024* | *SP1024* |
| ATP-dependent DNA helicase PcrA | *SP1087* | *SP1087* |
| Ribose-phosphate pyrophosphokinase | *SP1095* | *SP1095* |
| Ribonuclease HII | *SP1156* | *SP1156* |
| Ribonucleoside-diphosphate reductase 2 | *SP1180* | *SP1180* |
| DNA gyrase subunit A^a^ | *SP1219* | *SP1219* |
| L-lactate dehydrogenase | *SP1220* | *SP1220* |
| Amino acid ABC transporter, ATP-binding protein | *SP1242* | *SP1242* |
| Conserved hypothetical protein | *SP1247* | *SP1247* |
| DNA topoisomerase I | *SP1263* | *SP1263* |
| Carbamoyl-phosphate synthase, large subunit | *SP1275* | *SP1275* |
| Glucose-inhibited division protein B | *SP1285* | *SP1285* |
| Signal recognition particle protein | *SP1287* | *SP1287* |
| Cof family protein | *SP1291* | *SP1291* |
| Alanyl-tRNA synthetase | *SP1383* | *SP1383* |
| UDP-N-acetylenolpyruvoylglucosamine reductase | *SP1390* | *SP1390* |
| Hpr(Ser) kinase/phosphatase | *SP1413* | *SP1413* |
| ATP synthase F1, alpha subunit | *SP1510* | *SP1510* |
| Asparaginyl-tRNA synthetase | *SP1542* | *SP1542* |
| Triosephosphate isomerase | *SP1574* | *SP1574* |
| Adenine phosphoribosyltransferase | *SP1577* | *SP1577* |
| Sugar ABC transporter, ATP-binding protein | *SP1580* | *SP1580* |
| Ribosomal protein S15 | *SP1626* | *SP1626* |
| Recombination protein RecR | *SP1672* | *SP1672* |
| DNA-directed RNA polymerase, omega subunit | *SP1737* | *SP1737* |
| Thioredoxin | *SP1776* | *SP1776* |
| Alcohol dehydrogenase, zinc-containing | *SP1855* | *SP1855* |
| Oligopeptide ABC transporter, ATP-binding | *SP1887* | *SP1887* |
| Conserved hypothetical protein | *SP1922* | *SP1922* |
| Conserved hypothetical protein TIGR00150 | *SP1944* | *SP1944* |
| Hypothetical protein | *SP1962* | *SP1962* |
| UDP-N-acetylglucosamine 1-carboxyvinyltransferase | *SP1966* | *SP1966* |
| Transcription antitermination protein Nus | *SP2007* | *SP2007* |
| Tyrosyl-tRNA synthetase | *SP2100* | *SP2100* |
| Ribosomal protein L33 | *SP2135* | *SP2135* |
| ABC transporter, ATP-binding protein | *SP2220* | *SP2220* |
| ABC transporter, ATP-binding protein | *SP2221* | *SP2221* |
| Tryptophanyl-tRNA synthetase | *SP2229* | *SP2229* |

^a^ Previously identified genes
